# Inducing drop to bubble transformation via resonance in ultrasound

**DOI:** 10.1038/s41467-018-05949-0

**Published:** 2018-09-11

**Authors:** Duyang Zang, Lin Li, Wenli Di, Zehui Zhang, Changlin Ding, Zhen Chen, Wei Shen, Bernard P. Binks, Xingguo Geng

**Affiliations:** 1 Functional Soft Matter & Materials Group, Key Laboratory of Space Applied Physics and Chemistry of the Ministry of Education, School of Science, Northwestern Polytechnical University, Xi’an, 710129 China; 2 School of Mechanics, Civil Engineering and Architecture, Northwestern Polytechnical University, Xi’an, 710129 China; 3 Department of Chemical Engineering, Monash University, Wellington Road, Clayton, VIC 3800 Australia; 4 School of Mathematics and Physical Sciences, University of Hull, Hull, HU6 7RX UK

## Abstract

Bubble formation plays an important role in industries concerned with mineral flotation, food, cosmetics, and materials, which requires additional energy to produce the liquid–gas interfaces. A naturally observed fact is, owing to the effect of surface tension, a bubble film tends to retract to reduce its surface area. Here we show a “reverse” phenomenon whereby a drop is transformed into a bubble using acoustic levitation via acoustic resonance. Once the volume of the cavity encapsulated by the buckled film reaches a critical value V^*^, resonance occurs and an abrupt inflation is triggered, leading to the formation of a closed bubble. Experiments and simulations both reveal that V^*^ decreases with increasing acoustic frequency, which agrees well with acoustic resonance theory. The results afford enlightening insights into acoustic resonance and highlight its role in manipulating buckled fluid–fluid interfaces, providing a reference for fabricating unique core–shell-like materials.

## Abstract (web summary)

Bubbles tend to burst and retract into droplets. Here the authors show how a droplet can be turned into a bubble by levitating the droplets acoustically and exploiting their buckling to form gas bubbles.

## Introduction

As they float and burst, not only do soap bubbles amuse children with their iridescent coloring and whimsical nature, they also capture the interest of scientists wishing to investigate the underlying physics and chemistry^1–4^. Bubble formation plays an important role in the preparation of foams, which have extensive applications in industries concerned with food, cosmetics, pharmaceuticals, ultra-light materials, and mineral flotation^5^. Common approaches to forming bubbles are to exert intense shear to the liquid via turbulent mixing or flow focusing techniques^6^ or to use microfluidics^7^. In addition, surfactants or solid particles are typically introduced to reduce the surface energy of the gas–liquid interface and enhance the interfacial stability^8^. Because of the intrinsically high specific interfacial area of a soap bubble which is formed by a thin film, it is inevitable that a hole punctured in the film will grow either linearly for non-viscous films^9–11^ or exponentially for viscous films^12^. The bubble film retracts from the hole to reduce its surface area, either shattering into droplets as it bursts or forming daughter bubble cascades^2^.

In this work, the drop shape evolution and bubble formation are studied via ultrasonic levitation, which is often used in studies of droplet dynamics^13^ and manipulation^14,15^. By adjusting the sound intensity or sound field distribution, the shape of the acoustically levitated drop can be conveniently changed. It has been reported the levitated film can be buckled by the sound field and bubble formation phenomena have been evidenced by Lee et al.^16^ and Pathak and Basu^17^. The acoustic levitation technique provides the possibility to transform a liquid droplet into a bubble, however, the underlying mechanism is not clearly understood yet.

Here, we demonstrate that the bubble formation can be trigged in a controlled manner. Essential for this phenomenon is a buckled geometry that allows air to be encapsulated by the liquid film, thereby forming a resonance cavity, which has been verified by both experiments and numerical simulation. Once a critical cavity volume is achieved following significant buckling, the cavity resonates with the ultrasonic field leading to an abrupt increase in the cavity volume and rapid bubble formation. The insights presented herein shed light on the acoustic curving and manipulation of other fluid–fluid interfaces, such as the interface between a liquid drop or a gas bubble with an immiscible bulk liquid medium, providing a reference for fabricating unique soft materials, such as core–shell droplets^18^ and anti-bubbles^19^.

## Results

### Drop-to-bubble transition phenomenon in acoustic levitation

**Fig. 1:**
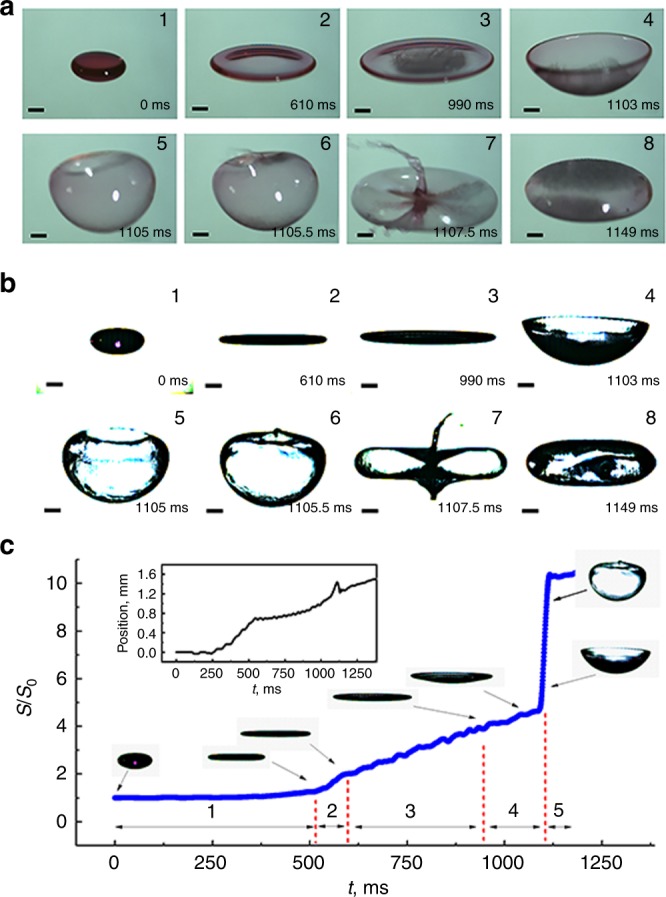
Drop-to-bubble transition of acoustically levitated drop. The process was triggered via increasing sound intensity through decreasing the emitter–reflector distance at a rate *u*_R_ = 1.50 mm/s. **a** Snapshots (taken with a high-speed camera titled at an angle of ~35°) of the evolution of a levitated oblate drop (0 ms) of aqueous sodium dodecyl sulfate (SDS) solution at its critical micelle concentration, CMC (~2.3 g/L). Upon increasing the sound intensity, the drop flattens (610 ms) and buckles (990 ms). The buckled liquid film then expands and its rim retracts inward (1103–1105.5 ms), forming a closed bubble (1149 ms). Liquid jetting is shown in 1107.5 ms. To enhance visibility, the drop was dyed with a commercial red ink. The volume of the drop is 10 μL. Each scale bar represents 1 mm. **b** Side-view snapshots of the same process as shown in **a**. **c** Surface area (*S*) variation of the drop with time divided into five stages: (1) slight deformation, (2) rapid flattening, (3) slow flattening, (4) buckling, and (5) abrupt expansion with rim closure. Inset photos show side-view snapshots corresponding to each stage. Inset graphics shows the levitation position (the initial drop centroid was defined as zero) of the drop/bubble was uplifted slightly (~1.5 mm) because of the lift of the nodal plane caused by the decrease of the emitter–reflector distance. The surface area is scaled to the initial surface area (*S*_0_) of a spherical drop

A typical drop-to-bubble transition is illustrated in Fig. 1. A drop of aqueous sodium dodecyl sulfate (SDS) was levitated at one of the nodal planes in a single-axis acoustic levitator comprising an emitter and a reflector both aligned vertically^20,21^, with the reflector fixed on a micro-lifting table. When the sound intensity was increased by decreasing the emitter–reflector distance at a controlled speed^22^, the levitated drop was deformed from an oblate spheroid into a thin film by the acoustic radiation force, which corresponds to integrating the acoustic radiation pressure on the drop surface^20^. As the liquid film moved upward in the ultrasonic field, it buckled before expanding and curving to form a bowl shape. The film eventually formed a closed air bubble with an oblate spheroidal shape similar to the initial drop, although possessing a much larger volume (Supplementary Movies 1 and 2). The ellipsoidal shape was determined by the acoustic radiation pressure exerted on the bubble surface which is negative (suction effect) at the equator area but positive (compression effect) at the polar regions^14,23^. It should be noted the shape (aspect ratio) of the obtained bubble can be adjusted by tuning the sound intensity through changing the emitter–reflector distance after the accomplishment of the drop-to-bubble transition (Supplementary Figure 1).

When the film was flattened sufficiently thin, capillary waves were excited^24^, which formed the interference patterns at the center of the film (Fig. 1a, 990 ms). The rim of the buckled film had a diameter larger than the thickness of the film lamella (Fig. 1a, 1103 ms), which is consistent with prior experimental observations that the extremely flattened droplet (610 ms in Fig. 1a) has a “dog bone-like” meridional cross section^22^. Since the enclosing rate of the film rim was very fast (~3 m/s), liquid jetting was often observed when it closed (1107.5 ms of Fig. 1a, b). Capillary waves could still be observed on the bubble surface (1149 ms, Fig. 1a, b).

### Time-evolution of drop surface area

To better understand the dynamics of the drop-to-bubble transition, we analyzed the time-dependent surface area of the drop/film. As the sound intensity was increased, the surface area (*S*) variation of the aqueous SDS drop was clearly divisible into five different stages: (1) slight deformation, (2) rapid flattening, (3) slow flattening, (4) buckling and finally (5) abrupt expansion with rim closure (Fig. 1c). The area of the liquid film increased very sharply between the end of stage 4 and into stage 5, indicating the onset of the drop-to-bubble transition. The levitation position was uplifted slightly upon the transition (inset graphics, Fig. 1c) owing to the lift of the nodal plane caused by the decrease of the emitter–reflector distance. It is unsurprising that the drop surface area increased continuously with sound intensity, because the drop assumes its equilibrium shape for any given sound pressure^25^. The abrupt area expansion in stage 5 was particularly interesting because it could not be explained by either static shape theory for acoustically levitated liquid droplets^14^ or by droplet instability theory^26^.

### Buckling of the acoustically levitated film

**Fig. 2:**
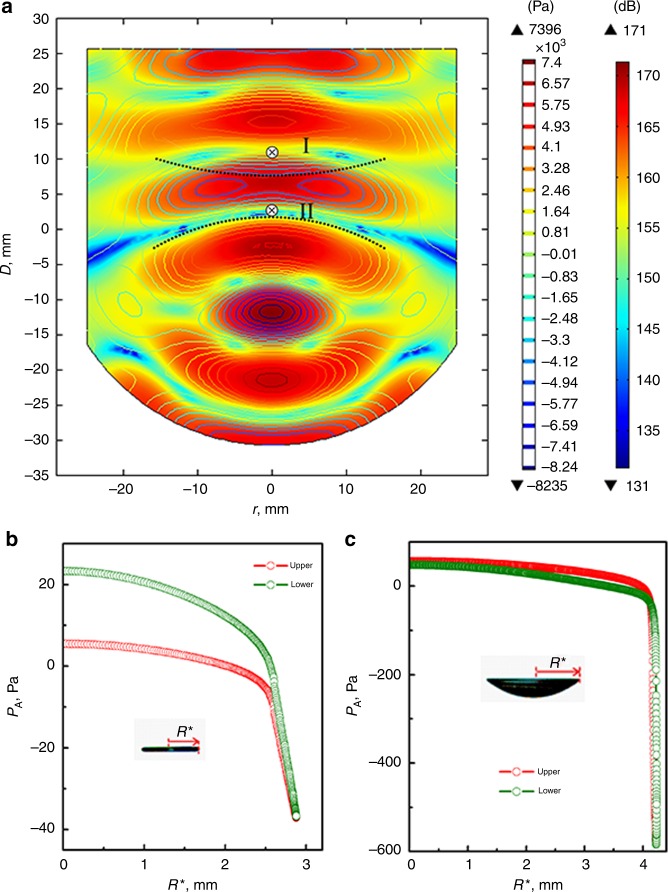
Sound field and acoustic radiation pressure calculation. **a** Distribution of sound pressure field in levitator with no levitated sample. Distance between emitter and reflector *D* is 40.0 mm corresponding to the initiation of liquid film buckling and *r* is the cross-sectional radius of the reflector. Sound pressure level at the center of the emitter surface is 155.7 dB. Black cross denotes the sound pressure nodes of the sound field, i.e. possible levitation positions under zero gravity. Levitation positions under normal gravity are below the crosses. Dotted lines indicate curvatures of equipotential surfaces close to positions I and II. **b** and **c** Acoustic radiation pressure *P*_A_ exerted on the liquid film surfaces (aqueous SDS drop, 10 μL) as a function of R* shown in inset: **b** flat liquid film, showing that *P*_A_ is larger on the lower surface, **c** buckled liquid film, showing that *P*_A_ on the upper surface becomes greater and is therefore responsible for the enhanced buckling

The buckling of the liquid film (Fig. 1c, stage 4) is one of the key features before the onset of the abrupt area expansion, which can be observed only if the film buckles sufficiently. To understand the physical mechanism of buckling, we numerically calculated the sound pressure field between the emitter and reflector; this distribution depends strongly on the reflector geometry which plays an essential role in enhancing levitation ability and stability^20,27^. Fig. 2a illustrates the original sound pressure distribution in the levitator before the levitation sample was positioned. Because of the curved shape of the reflector, the equipotential surfaces of the sound field were not planar and thus represented the buckling direction qualitatively. The equipotential surface was concave near position I but convex near position II, as guided by the dotted lines in Fig. 2a, which is consistent with the fact that the liquid film buckled upward when levitated at position I but downward at position II (Supplementary Movie 3). This suggests that the flattened thin film tends to follow the equipotential surface^28,29^. We also found that the buckling direction reversed when the levitator was inverted (Supplementary Movie 4), implying that gravity was negligible in determining the buckling direction. These experiments showed clearly that the preferred buckling direction was set by the equipotential surface in the sound field of the levitator.

To gain a deeper insight into the buckling behavior of the levitated liquid film, the acoustic radiation pressure *P*_A_ on the sample surface was calculated based on King’s theory:^30^

1 $$P_{\mathrm{A}} = \frac{1}{{2\rho _0c_0^2}}\left\langle {p^2} \right\rangle - \frac{1}{2}\rho _0\left\langle {v^2} \right\rangle$$

where *p* is the sound pressure, *c*_0_ is sound speed, *ρ*_0_ is the density of air, and *v* is the particle (parcel of fluid) velocity of the medium. When the liquid film was flat, *P*_A_ on its lower surface was larger than that on its upper surface (Fig. 2b), indicating that the drop was pushed upward by the ultrasound to balance the effect of gravity. Once the drop had buckled, *P*_A_ on the upper surface became dominant (Fig. 2c) and now the effect of gravity had to be balanced by the suction effect (negative *P*_A_) at the rim. The difference ∆*P*_A_ in acoustic radiation pressure between the upper and lower surfaces buckled the liquid film and enhanced the buckling with increasing sound intensity. It should be noted that the suction effect at the rim of the liquid film was strengthened significantly after buckling (Fig. 2c).

### Inflation of the cavity encapsulated by the buckled film

**Fig. 3:**
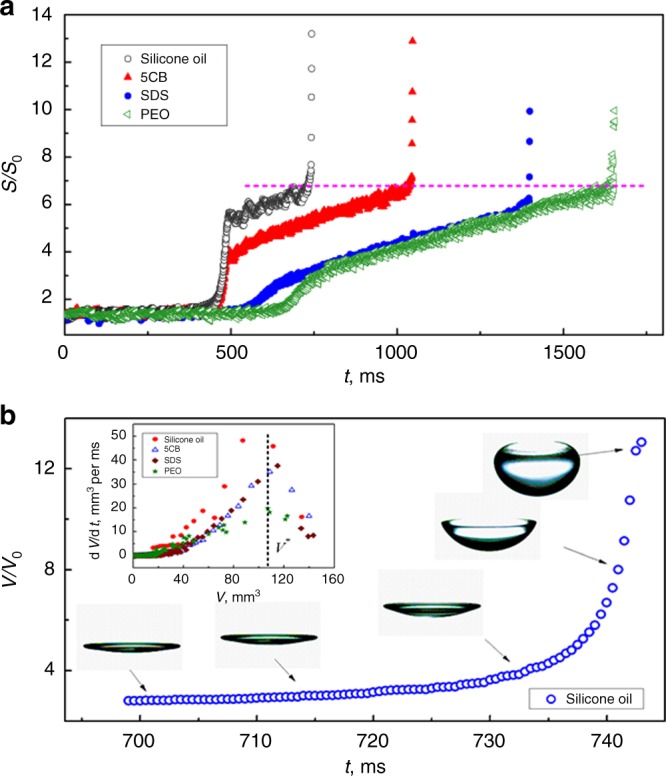
Surface area variation and cavity inflation during bubble formation. The sound intensity was increased at *u*_R_ = 1.00 mm/s. **a** Surface area (normalized by the surface area of a spherical drop) variation for drops of different liquids with similar five stage behavior. The abrupt area expansion occurs at different time intervals, i.e. emitter–reflector distance, but corresponds to similar surface area marked by the dashed line. **b** Cavity volume of silicone oil (normalized by the initial drop volume) as a function of time. The inset graph shows that the maximum inflation rate, d*V*/d*t*, corresponds to the same critical volume V* for all the liquids

Similar shape evolution and surface area variation stages have been observed for other liquids (aqueous and non-aqueous) upon decreasing the emitter–reflector distance at a rate of 1.00 mm/s (Fig. 3a). However, the duration needed (i.e. the emitter–reflector distance) to induce the transition is highly dependent on the system; liquids of higher surface tension require higher sound intensities to initiate the transition. This is because one of the crucial factors to trigger the transition is film buckling which requires a Laplace pressure ∆*P*_L_ ~ 4*σ*/*R*_B_ (where *σ* is the liquid surface tension, *R*_B_ is the radius of curvature) to be provided by the sound field. Interestingly however, the abrupt area expansion of all the liquid occurs at almost the same surface area (Fig. 3a). It should be noted that an air cavity was formed once the liquid film buckled. With the enhancement of buckling, the volume *V* of the cavity encapsulated by the buckled film increases as well (Fig. 3b). Upon increasing the sound intensity, an abrupt inflation of the open cavities was observed for all liquids (Supplementary Figure 2). However, the maximum inflation rate (d*V*/d*t*) for all liquids corresponds to a very similar cavity volume V^*^ (Fig. 3b, inset). This suggests that the cavity volume played a crucial role in the drop-to-bubble transition.

### Acoustic resonance mechanism

It remains to be explained why abrupt inflation of the open cavity, i.e. the maximum in d*V*/d*t*, corresponds to the same cavity volume. Based on acoustic resonance theory^31^, the air cavity of volume *V* with an opening of diameter *d* can be regarded as a Helmholtz resonator with a resonant frequency *f* determined by its geometry. This is analogous to an inductor–capacitor circuit^32^. The air cavity acts as the capacitor with capacitance $C_0 = V/(\rho _0c_0^2)$ and the opening is the inductor with inductance $L_0 = \rho _0d_{\mathrm {{eff}}}/S_{\mathrm {{h}}}$, where *S*_h_ is the area of the opening and *d*_eff_ is the effective depth of the cavity ($d_{\mathrm {{eff}}} = t + 1.8\sqrt d$, *t* being the thickness of the liquid film). Therefore, the resonant frequency *f* of such a resonator can be written as^33^

2 $$f = \frac{1}{{2\pi \sqrt {L_0 \cdot C_0} }} = \frac{{c_0}}{{2\pi \sqrt {\frac{{Vd_{\mathrm {{eff}}}}}{{S_{\mathrm {h}}}}} }}$$

This indicates that once the air cavity achieves an appropriate volume through buckling, it may resonate with the sound field of the levitator and significantly enhance energy adsorption. In this case, the air molecules inside the cavity vibrate intensely thus leading to a high sound pressure, which results in abrupt cavity inflation and bubble formation.

**Fig. 4:**
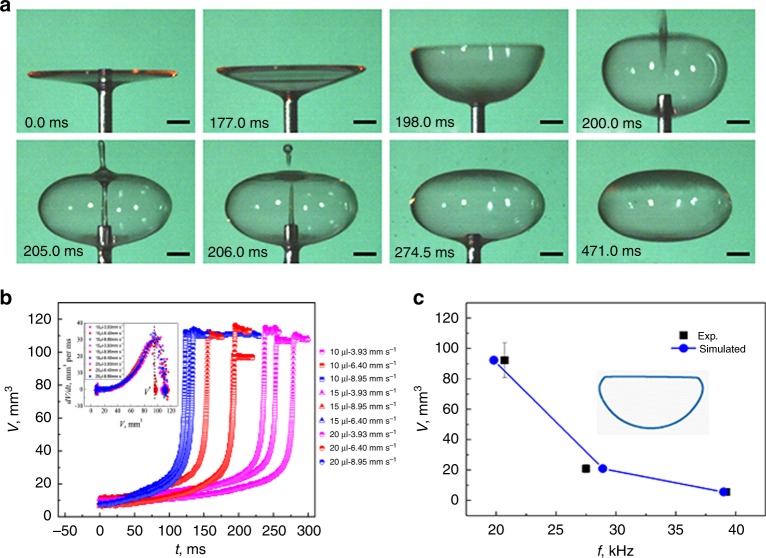
Resonance mechanism for bubble formation of acoustically levitated drops. All the drops were SDS drops at CMC. **a** Snapshots showing that dragging a needle positioned at the center of the film caused it to buckle, resulting in abrupt inflation and bubble formation. Each scale bar represents 1 mm. **b** Cavity volume *V* as a function of dragging time for liquid films of different initial volumes (10, 15 and 20 µL) and different dragging rates (3.93–8.95 mm/s). Similar abrupt volume inflation was observed corresponding to the same critical cavity volume V^*^ (see inset). **c** V^*^ as a function of working frequency of the levitator; squares are experiment, circles are simulation. Inset image illustrates the contour line of the cavity corresponding to the maximum inflation rate for 20.7 kHz

To gain a quantitative understanding of the abrupt inflation and bubble formation phenomenon, the buckling degree of the liquid film was set in a controlled manner by dragging its center using a needle. A similar bubble formation process was observed (Fig. 4a, Supplementary Movie 5). Note that dragging with a needle only gives the liquid film its initial buckled shape and hence cavity volume; the subsequent abrupt area expansion, closure and bubble formation are driven by the sound field and show no significant difference to the sequence shown in Fig. 1. The maximum inflation rate d*V*/d*t* was evidenced at the same critical cavity volume V^*^ and was independent of the initial liquid volume and dragging rate (Fig. 4b, inset). However, V^*^ depends strongly on the working frequency of the levitator (Fig. 4c). The resonance frequency for the cavity with the same geometry extracted from images taken by high speed camera was simulated based on acoustic resonant theory^33^, which agrees well with the experimental results (Fig. 4c and Supplementary Figure 3). With the enhancement of buckling, i.e. cavity volume, the energy absorption of the cavity from the sound field becomes more significant until the occurrence of resonance, which is reflected in the sound pressure distribution inside the cavity (Supplementary Figure 4). The results confirmed it was the resonance mechanism that dominates the abrupt inflation and bubble formation with an acoustically levitated buckled liquid film.

**Fig. 5:**
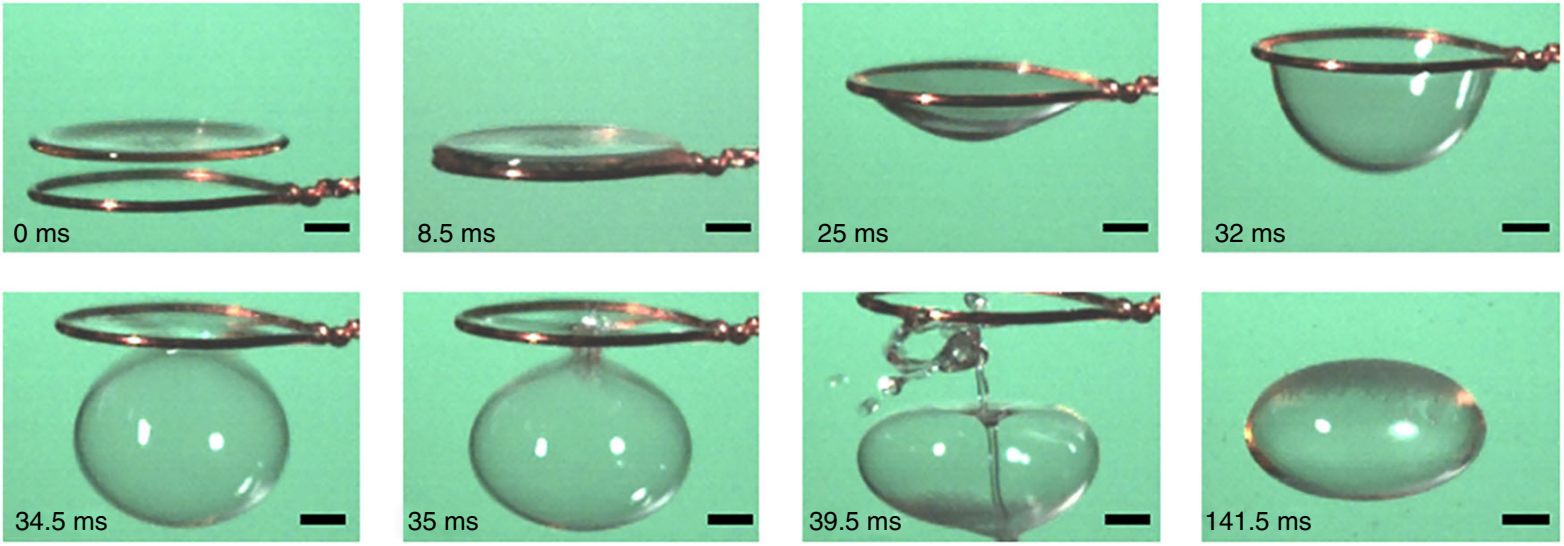
Bubble formation of a liquid film buckled via a rigid ring. Snapshots showing that bubble formation can be triggered by dragging a ring of metal wire constantly around the edge of the film (SDS drops at CMC), which completely limited the radial oscillation of the levitated sample. Scale bars represent 1 mm

Alternatively, buckling could be caused by dragging a ring of metal wire from the edge of the liquid film triggering similar bubble formation (Fig. 5, Supplementary Movie 6). In this case, radial oscillations were completely inhibited. However, the surface area and cavity volume still expanded abruptly when a critical cavity volume was reached. This clarified that radial oscillations, which are often observed in acoustically levitated drops^34^, play little role in this bubble formation phenomenon.

## Discussion

The drop-to-bubble transition observed here undergoes similar drop deformation and exhibits similar bubble shapes as the “bag breakup” phenomenon of free falling raindrops^35^ or drops in a wind tunnel^36^. However, a large Weber number is not necessary in our case. Because of the difference in aerodynamic pressure inside and outside the “bag,” it often inflates without rim closure until bursting^37^, whereas in our work the bubble was formed via a resonance mechanism. Therefore, the acoustic energy can be adsorbed efficiently on a timescale of milliseconds and transferred into the energy of the bubble surface. In this case, the acoustic wave gives rise to a very unique approach to bubble fabrication, not only providing the levitation force against gravity but also affording the energy with which to produce new surface. In addition, the acoustic resonance mechanism may also be the origin of many other processes involving ultrasound, such as ultrasound foaming^38^ and emulsification^39^.

The final size of the obtained bubble is largely dependent on the work frequency of the levitator as indicated by Eq. (2) and our experimental observations (Fig. 4). The curvature of the reflector only influenced slightly the final bubble volume (Supplementary Table 1) due to the minor shape change in the sound potential well. This hints that it is possible to fabricate micron sized bubbles which are often used as ultrasound contrast agents^40^ by using MHz levitators. But at such high frequency, it would be hard to accomplish stable levitation in air because in a gaseous medium the attenuation for MHz sound waves becomes extremely significant^31^. Furthermore, acoustic streaming could seriously influence the levitation stability for droplets smaller than mm scale^41^. These facts suggest one of the potential applications of this technique is to perform acoustic resonance in liquid media.

It should be noted that acoustically levitated bubbles can last over tens of minutes without bursting, which is much more stable than the non-levitated soap bubble. The eventual collapse of the bubble may be caused by evaporation since acoustic levitation itself does not suppress evaporation. What is interesting is it provides a mechanism to significantly retard liquid drainage and leads to this extraordinary stability, although the underlying physics is required for further study.

In conclusion, the most important finding of this study is that the cavity encapsulated by the acoustically levitated buckled liquid film can be regarded as an acoustic resonator that is independent of the liquid properties. Once the cavity reaches an appropriate volume induced by increased liquid film buckling by either enhanced sound intensity or external dragging, acoustic resonance occurs and abrupt inflation is then triggered leading to bubble formation. Our results establish a unique bubble formation method and create an excellent platform for studying bubble physics, such as oscillation, drainage and evaporation. The technique also provides a reference for fabricating unique core–shell-like materials via the acoustic resonance mechanism.

## Methods

### Materials

The different liquids we used for acoustic levitation were aqueous solutions of SDS and poly(ethylene oxide) (PEO, molecular weight ~10^6^ amu), a liquid crystal (5CB), silicone oil PMX-200, and water. The water was purified with an Ultrapure Water System (EPED, China) and all other materials were purchased from Aladdin Industrial Corporation, China. The surface tension *σ* of the liquid was measured with a Wilhelmy plate using a Langmuir-trough instrument (JML04C3, Powereach Ltd., China). The viscosity *η* of the liquid was measured with a stress-controlled rheometer (Physica MCR 302; Anton Paar, Germany) equipped with cone-and-plate geometry (at typical shear rate range 10^2^–2 × 10^3^ s^−1^) and glass capillary viscometers. All the liquids were treated as Newtonian because the process before the onset of the drop-to-bubble transition was quasi-static. Detailed properties of the liquids are provided in Table 1.

**Table 1:** Parameters for different liquids used in the experiments at 25 °C

Liquid	Concentration (g/L)	Density (g/cm^3^)	Viscosity (mPa s)	Surface tension (mN/m)
Water	–	0.998	0.90	72.6
Aqueous SDS	2.3 (~cmc)	0.994	1.15	41.8
5CB (cyanobiphenyl)	–	1.008	40.30	35.9
Aqueous PEO	0.5	1.008	1.30	61.9
Silicone oil	–	0.963	100.00	21.0

### Experimental setup and procedure

The acoustic levitator was custom built and comprised an emitter and reflector arranged coaxially along the gravitational direction, as illustrated in Supplementary Figure 5. To study the effect of acoustic frequency, we used three different levitators operating at frequencies of 20.7, 27.5, and 39.2 kHz, providing both sufficient levitation capability and satisfactory stability.

To adjust the distance between the emitter and reflector conveniently, the reflector was fixed on a micro-lifting table (ST401ES60, Strong Precision, China). The lift rate *u*_R_ of the reflector could be controlled accurately with a servomotor (42BYGH47-1684B, Sihongmotor, China); we set *u*_R_ = 1.00 or 1.50 mm/s. The acoustically levitated liquid film could be buckled by external dragging with a needle or a circular frame, which were also controlled by a servomotor. The dragging rate was 3.93–8.95 mm/s. All the experiments were performed in a clean room at room temperature of ~25 °C and a relative humidity of ~40%.

### High speed camera and image analysis

The dynamics of the levitated droplets was recorded by two high-speed CCD cameras, namely CCD1 (Trouble Shooter HR, USA) and CCD2 (Photron Fastcam Mini UX100, Japan) at frame rates of 2000–10,000 fps. To understand the time variation of the surface area of the liquid film and the volume of the cavity encapsulated by the buckled film, the recorded videos were analyzed using MATLAB 2017 with an in-house compiled code. Each frame was transferred into a 256 gray-scale image via gray processing, where the gray value is 0 for black pixels and 255 for white pixels. The Gaussian low-pass filtering was selected to smooth the gray-scale image to suppress the noise. Then the gradient could be calculated based on the Gaussian filtering output. The boundaries of the drop or liquid film were extracted by detecting the local maximum of gray-scale gradient, as described by Canny^42^. Based on the determination of sample boundaries, the surface area could be regarded as the summation of circular stripes formed by each layer of pixels, while the volume could be treated as the total volume of cylinders surrounded by each layer of pixels. The surface area and cavity volume could then be calculated via the integral approach. The accuracy of the method was calibrated by using a standardized solid sphere which showed the error for area calculation is smaller than 3.0% and for volume calculation it is less than 0.5%.

### Simulations

The sound field in the levitator, the acoustic radiation pressure on the sample surface, and the acoustic resonant properties of the cavity were calculated using commercial finite-element software COMSOL Multiphysics 5.2a. For the calculation of sound field and acoustic radiation pressure, a two-dimensional axisymmetric model was employed. The simulation domain was determined by the geometry of the levitator where the reflector was configured as a rigid boundary and the side wall was set as the radiation boundary condition. In the levitator, the acoustic medium was air (density *ρ*_0_ = 1.18 kg/m^3^, sound velocity *c*_0_ = 346.12 m/s) and the liquid drop/film was configured as a continuity boundary. For simplicity, the material of the drop was assumed to be water (*ρ* = 998.2 kg/m^3^, sound velocity *c*_water_ = 1495.33 m/s) because the acoustic impedance mismatch between air and all the liquids used in the experiments were similar.

For the simulation of acoustic resonant properties, the cavity encapsulated by a water shell was placed in a waveguide tube filled with air (Supplementary Figure 6). The model of the cavity was obtained by rotation of the contour line of the sample extracted from high speed camera images. The side walls of the tube were set as rigid boundaries whereas the front and back faces were set as the “radiation boundary condition”. The entire simulated domain was meshed by the tetrahedron and the user pre-defined size was set as “Fine”. In the simulation, a 1-Pa amplitude plane harmonic wave was sent into the simulated domain via the radiation boundary condition at the front face of the waveguide, and a zero radiation boundary condition was applied to the back face. This enabled user-defined plane waves to enter into the simulation domain with all the incident waves being completely absorbed. The acoustic resonant properties can be derived by analyzing the transmission and acoustic field distribution in the tube. The sharp adsorption peak in the adsorption spectrum represents the minimum of energy transmission, which indicates the occurrence of resonance. The corresponding frequency of the adsorption peak is the resonance frequency.

### Data availability

The data that support the findings of this study are available from the corresponding author upon reasonable request.

## Electronic supplementary material

Supplementary Information

Description of Additional Supplementary Files

Supplementary Movie 1

Supplementary Movie 2

Supplementary Movie 3

Supplementary Movie 4

Supplementary Movie 5

Supplementary Movie 6
