# Supplementary material for: Inducing drop to bubble transformation via resonance in ultrasound
Source: Nat Commun. 2018 Sep 11;9:3546. doi: 10.1038/s41467-018-05949-0 (PMC6133948; doi:10.1038/s41467-018-05949-0)
Supplement: Supplementary file 1 — Supplementary Information [file 41467_2018_5949_MOESM1_ESM.pdf]

Supplementary Information

Inducing drop to bubble transformation *via* resonance in ultrasound

Duyang Zang,[1*] Lin Li,[2] Wenli Di,[1] Zehui Zhang,[2] Changlin Ding,[1] Zhen Chen,[1] Wei Shen,[3] Bernard P. Binks[4] and Xingguo Geng[1]

1 *Functional Soft Matter & Materials Group, Key Laboratory of Space Applied Physics and Chemistry of the Ministry of Education, School of Science, Northwestern Polytechnical University, Xi'an 710129, China*

2 *School of Mechanics, Civil Engineering and Architecture, Northwestern Polytechnical University, Xi'an 710129, China*

3 *Department of Chemical Engineering, Monash University, Wellington Road, Clayton, Victoria 3800, Australia*

4 *School of Mathematics and Physical Sciences, University of Hull, Hull. HU6 7RX. UK*

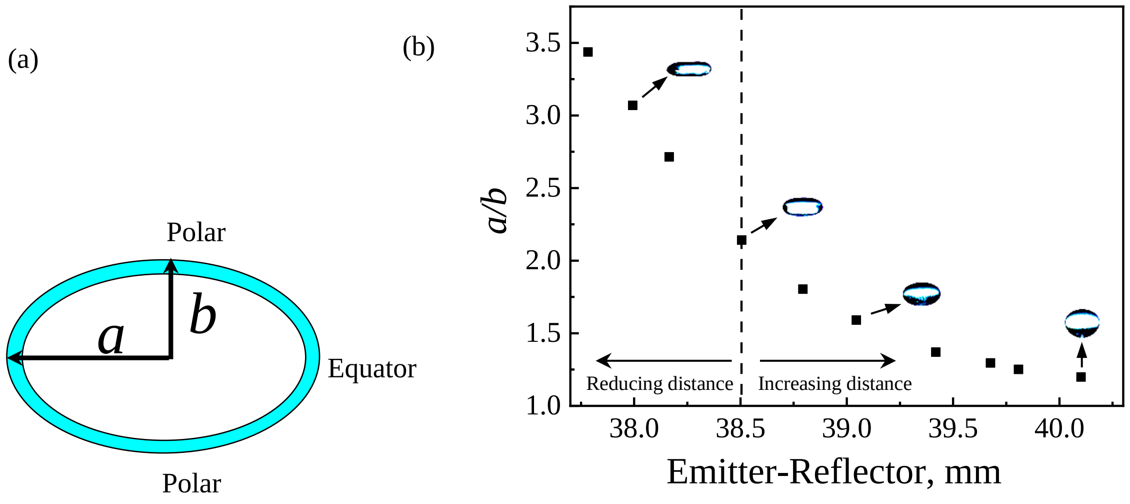

Supplementary Figure 1. Bubble shape versus sound intensity. (a) Schematic view of a bubble, (b) aspect ratio *a*/*b* of the bubble (obtained from 1xcmc SDS drop, 10 μL) as a function of emitter-reflector distance. The dotted line indicates the emitter-reflector distance corresponding to the initial drop-bubble transition.

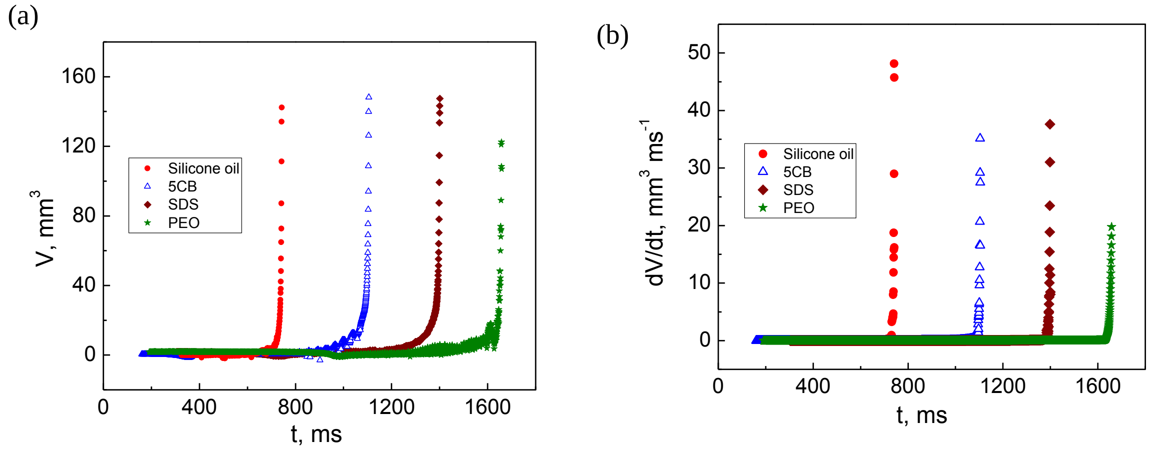

Supplementary Figure 2. Variation of (a) cavity volume V and (b) its inflation rate dV/dt as a function of time for drops of different liquids (10 μL) upon increasing the sound intensity by reducing the emitter-reflector distance at u_R = 1.00 mm s^{-1}.

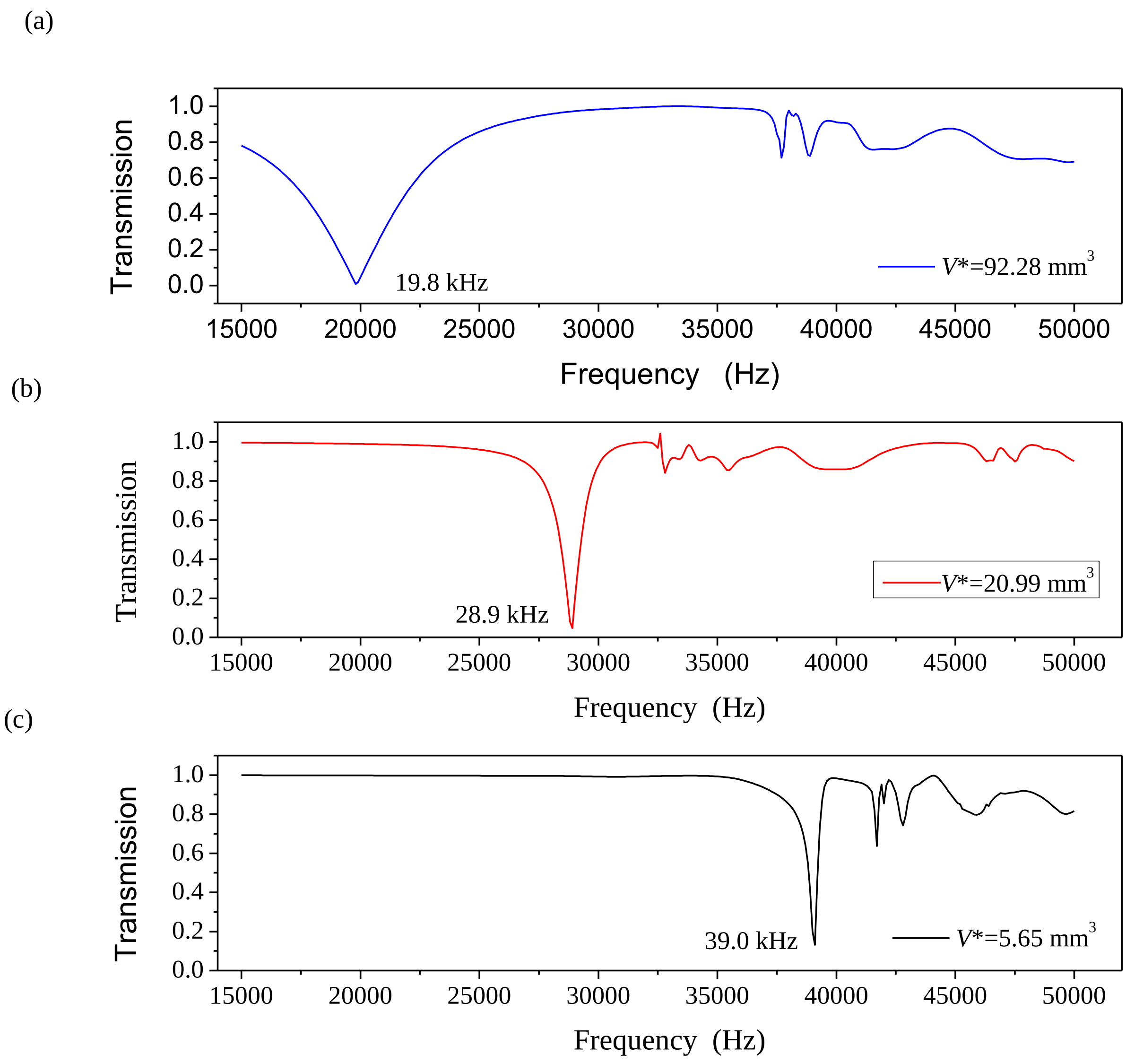

Supplementary Figure 3. Simulation of acoustic resonant properties of the air cavity encapsulated by the buckled liquid film. The model of the cavity is built by rotation of the contour line of the sample extracted from the images taken with the high speed camera, which possess the maximum inflation rate. (a), (b) and (c) correspond to the cavities in levitators of 20.7, 27.5 and 39.2 kHz respectively. The downward peak shows the maximum energy adsorption by the cavity from the sound field at a certain frequency indicating the resonance behavior.

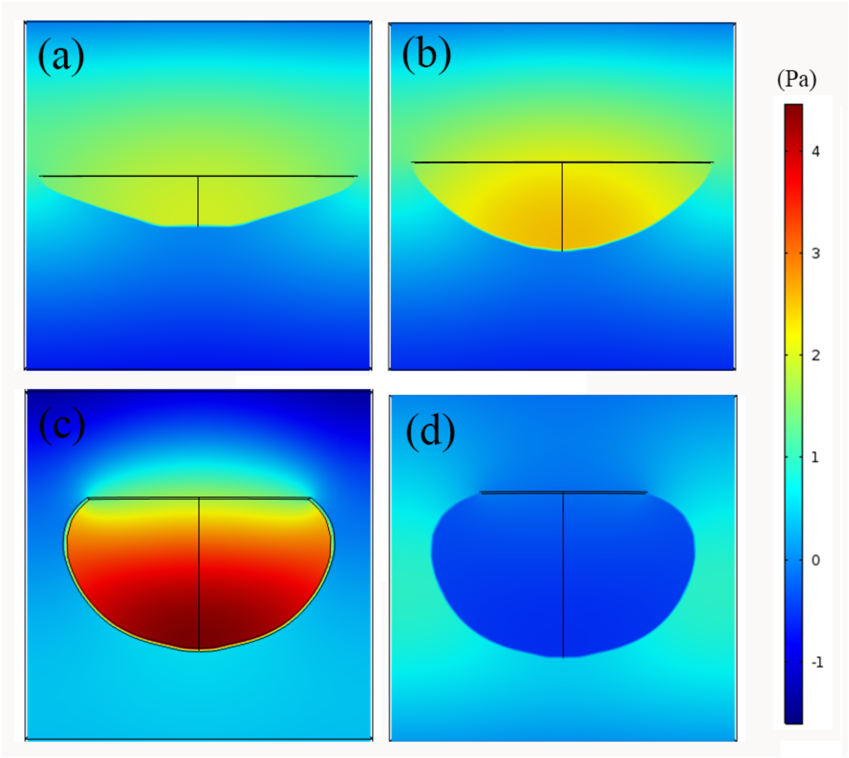

Supplementary Figure 4. Sound pressure distribution in the air cavities of varied volume corresponding to the shape evolution of a liquid film levitated in a 20.7 kHz levitator. The volumes for (a)-(d) are 30.01, 56.27, 92.28 and 106.76 mm^3 respectively. The cavity of (c) corresponds to the maximum inflation rate and also shows the strongest energy adsorption.

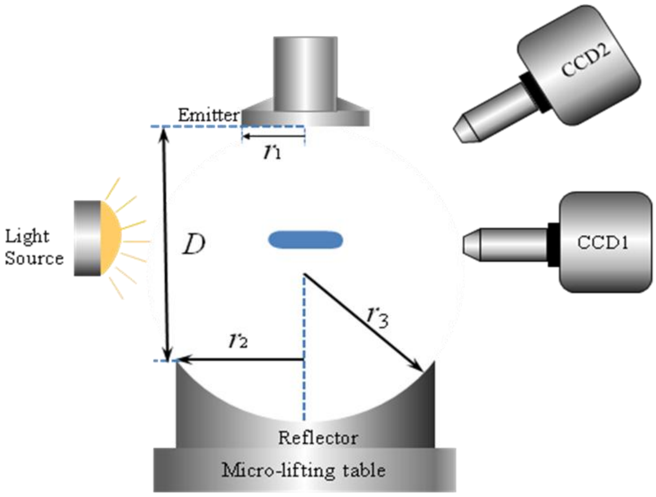

Supplementary Figure 5. Schematic representation of the experimental setup. r_1 = 12.5 mm, r_2 = 25.0 mm, r_3 = 37.4 mm.

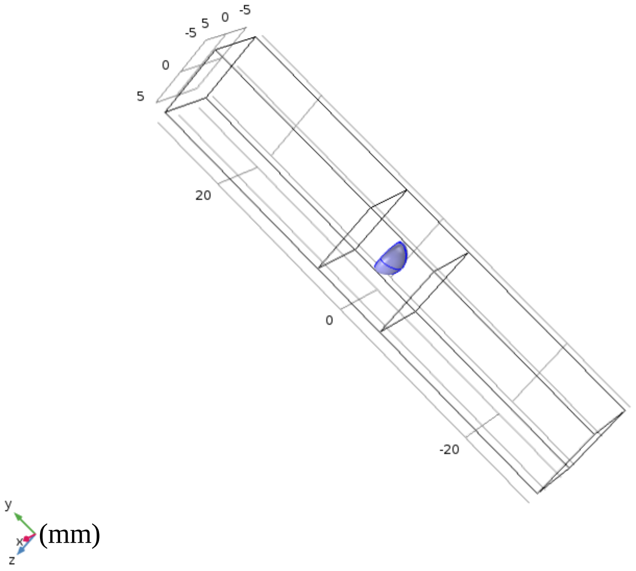

Supplementary Figure 6. Schematic illustration of the simulation domain and cavity model.

Supplementary Table 1. Final bubble volume influenced by the curvature of the reflector, where the bubble was obtained from SDS drops (10 μL) in a 20.7 kHz levitator, the corresponding Emitter-Reflector distance was ~ 28.5 mm.

Curvature radius of reflector (mm)	2a (mm)	2b (mm)	Final bubble volume (mm^3)
30.0	7.48	3.35	91.47
37.4	7.20	3.95	111.84
40.0	7.53	3.88	105.12
60.0	7.72	4.05	118.05
Flat (∞)	7.83	3.65	112.86
