# Supplementary material for: Inducing drop to bubble transformation via resonance in ultrasound
Source: Nat Commun. 2018 Sep 11;9:3546. doi: 10.1038/s41467-018-05949-0 (PMC6133948; doi:10.1038/s41467-018-05949-0)
Supplement: Supplementary file 2 — Description of Additional Supplementary Files [file 41467_2018_5949_MOESM2_ESM.pdf]

Description of Additional Supplementary Files

Supplementary Movie 1

Description: Droplet-to-bubble transition with increasing sound intensity of a SDS solution droplet (10 μl), with a high speed camera titled at an angle~35°.

Supplementary Movie 2

Description: Droplet-to-bubble transition with increasing sound intensity of a SDS solution droplet (10 μl), side view.

Supplementary Movie 3

Description: SDS solution droplet (10 μl) buckles downwards with increasing sound intensity at levitation position Ⅱ.

Supplementary Movie 4

Description: SDS solution droplet (10 μl) buckles downwards with increasing sound intensity at levitation position Ⅰ when the levitator is inverted.

Supplementary Movie 5

Description: Bubble formation with an acoustically levitated liquid film buckled via dragging with a needle from the center of the film.

Supplementary Movie 6

Description: Bubble formation with an acoustically levitated liquid film buckled by dragging a ring of metal wire from the edge of the film.
